# Applying hiPSCs and Biomaterials Towards an Understanding and Treatment of Traumatic Brain Injury

**DOI:** 10.3389/fncel.2020.594304

**Published:** 2020-11-12

**Authors:** María Lacalle-Aurioles, Camille Cassel de Camps, Cornelia E. Zorca, Lenore K. Beitel, Thomas M. Durcan

**Affiliations:** ^1^Early Drug Discovery Unit, Montreal Neurological Institute-Hospital, McGill University, Montreal, QC, Canada; ^2^Department of Biological and Biomedical Engineering, McGill University, Montreal, QC, Canada

**Keywords:** hiPSC, regenerative therapies, stem cells, biomateriais, traumatic brain injury

## Abstract

Traumatic brain injury (TBI) is the leading cause of disability and mortality in children and young adults and has a profound impact on the socio-economic wellbeing of patients and their families. Initially, brain damage is caused by mechanical stress-induced axonal injury and vascular dysfunction, which can include hemorrhage, blood-brain barrier disruption, and ischemia. Subsequent neuronal degeneration, chronic inflammation, demyelination, oxidative stress, and the spread of excitotoxicity can further aggravate disease pathology. Thus, TBI treatment requires prompt intervention to protect against neuronal and vascular degeneration. Rapid advances in the field of stem cells (SCs) have revolutionized the prospect of repairing brain function following TBI. However, more than that, SCs can contribute substantially to our knowledge of this multifaced pathology. Research, based on human induced pluripotent SCs (hiPSCs) can help decode the molecular pathways of degeneration and recovery of neuronal and glial function, which makes these cells valuable tools for drug screening. Additionally, experimental approaches that include hiPSC-derived engineered tissues (brain organoids and bio-printed constructs) and biomaterials represent a step forward for the field of regenerative medicine since they provide a more suitable microenvironment that enhances cell survival and grafting success. In this review, we highlight the important role of hiPSCs in better understanding the molecular pathways of TBI-related pathology and in developing novel therapeutic approaches, building on where we are at present. We summarize some of the most relevant findings for regenerative therapies using biomaterials and outline key challenges for TBI treatments that remain to be addressed.

## Introduction

Traumatic brain injury (TBI) is defined as a disruption in the normal function of the brain caused by a sudden blow or jolt to the head, which is frequently suffered during unintentional falls, sporting activities, automotive accidents, or violent assaults (Peterson and Kegler, 2020). According to the World Health Organization, TBI affects 69 million individuals globally, with the highest prevalence in North America and Europe (Dewan et al., 2019), where it has become the leading cause of disability and mortality in children and young adults. The mechanical force-driven axonal damage and vascular dysfunction that occur during the acute phase of TBI are followed by chronic inflammation, oxidative stress, and cytotoxicity. These effects extend and aggravate neurodegeneration and vascular pathology over time (Masel and DeWitt, 2010). Thus, TBI is not a one-time event; rather it resembles a chronic degenerative disease. This progressive deterioration of the brain, in addition to causing mental and physical disability at early stages, increases the risk of further developing Alzheimer’s disease, Parkinson’s disease, chronic traumatic encephalopathy, and sporadic amyotrophic lateral sclerosis (VanItallie, 2019). Therefore, preventing and mitigating the outcomes of TBI is a priority for the health care system.

Clinical therapies have predominantly targeted brain edema, oxidative stress, and inflammation, but despite decades of focused research, pharmacological and non-pharmacological interventions aimed at improving the quality of life for patients with TBI are scant (reviewed in Kochanek et al., 2015). Regenerative medicine, based on the potential of stem cells (SCs) to repair neuronal and vascular damage, has emerged as a promising therapeutic strategy. However, clinical translation of these therapies remains limited (Hasan et al., 2017; Reis et al., 2017; Bonsack et al., 2020). A deeper understanding of the proliferation and differentiation capabilities of different SCs, the optimal administration routes, their interplay with other tissue-resident cells, and potential side effects is essential for advancing stem cell-based therapies for TBI (Dekmak et al., 2018). On the other hand, since neurons are highly dependent on nutrients and oxygen supply from the vasculature and on the homeostatic activity of astrocytes and microglia, it is important to restore the full environment for these therapies to succeed. However, the vast majority of research in the field has focused on restoring neuronal function by promoting neurogenesis and often disregard other facets of the degenerative process that are equally relevant such as the recovery of the brain vasculature and the glial cell function. Here, we review the multifaced degenerative processes linked to TBI, focusing on how stem cell research, namely through the use of human induced pluripotent stem cells (hiPSCs; Takahashi et al., 2007), can help decipher the molecular pathways suitable for pharmacotherapy and facilitate drug screening in the context of personalized medicine. HiPSCs are derived from somatic cells such as fibroblasts or peripheral blood mononuclear cells (PBMC) through the Yamanaka reprogramming factors (Oct4, Sox2, c-Myc, and Klf4; Takahashi and Yamanaka, 2006; Takahashi et al., 2007). These cells present with similar characteristics as those of human embryonic stem cells (hESCs) for both morphology, proliferation, surface antigens, gene expression, and epigenetic status of pluripotent cell-specific genes with the advantage of alleviating ethical concerns about their use for biomedical research and clinical therapies (Chang et al., 2019; Seranova et al., 2020).

We also describe how engineered tissues (i.e., brain organoids, bio-printed tissues, and biomaterials) can enhance the success of stem cell therapies in the treatment of TBI.

## Molecular Basis of TBI-Induced Neuronal Dysfunction and Repair Pathways

### Diffuse Axonal Injury, White Matter Degeneration, and Repair

Diffuse axonal injury (DAI) caused by shear stress is one of the first histopathological hallmarks of brain trauma. It is particularly evident in the gray matter (GM)–white matter (WM) interface due to the marked transition between these two tissues with different deformation properties (Sharp et al., 2014; Armstrong et al., 2016). Axonal injury substantially disrupts the intrinsic connectivity networks, precipitating a cognitive decline in TBI patients (Sharp et al., 2014). Moreover, in subsequent hours or even days after injury, axons often undergo the process of Wallerian degeneration (Wld), resulting in axonal loss and WM atrophy (Koliatsos and Alexandris, 2019). Thus, Wld prevention has become one of the principal targets for therapies against neurodegeneration in TBI.

Wld is thought to be mediated by severe deprivation of nicotinamide adenine dinucleotide (NAD^+^), a redox cofactor essential for axonal maintenance that plays an important role in protecting axons from mechanical injuries and ischemia (Fricker et al., 2018). Following DAI, activation of the stress mitogen-activated protein kinase (MAPK) pathway induces the depletion of nicotinamide mononucleotide adenylyltransferase 2 (NMNAT2), compromising NAD^+^ biosynthesis (Walker et al., 2017). In parallel, NMNAT2 loss activates the sterile α and Toll/interleukin-1 receptor (TIR) motif-containing 1 (SARM1) domain, which has intrinsic NADase enzymatic activity, thus drastically impacting NAD^+^ availability and triggering neuronal death (Coleman and Freeman, 2010; Gerdts et al., 2016; Brazill et al., 2017; Essuman et al., 2017; Koliatsos and Alexandris, 2019). Downregulation of SARM1 and upregulation of NMNAT2 are promising strategies for Wld prevention in TBI, but the specific mechanisms for SARM1-NMNAT2 interactions have yet to be elucidated to enhance drug efficiency and ensure neuroprotection (Ziogas and Koliatsos, 2018).

Small molecule screening for modulators of NMNAT2 activity has identified compounds that enhance neuroprotection in mouse primary neuronal cultures (Ali et al., 2017). Similarly, SARM1 deletion successfully reduced axonal damage, demyelination, and WM atrophy in TBI mouse models (Marion et al., 2019). Most promisingly, pharmacological blockade of the SARM1 NADase enzymatic activity, responsible for axonal degeneration, with small-molecule inhibitors has proven beneficial in protecting hiPSC-derived motor neurons from traumatic injury (Krauss et al., 2019, 2020). The work by Krauss and collaborators demonstrates that pharmacological inhibition of the SARM1 enzymatic activity in mechanically injured hiPSC-derived motor neurons mimics the axonal protective phenotype (reduced axonal fragmentation post-injury) observed in SARM1 knockout mice. In this regard, the possibility of performing high-content drug screening on patient-derived iPSC represents a major advance towards precision medicine in the field of TBI and provides an excellent tool to decode the molecular mechanisms of axonal protection and regeneration in the human genetic background.

In addition to axonal injury, oligodendrocytes, whose main role is to maintain myelin sheaths and provide trophic support for axons (Baumann and Pham-Dinh, 2001; Du and Dreyfus, 2002; Morrison et al., 2013), are also subject to degeneration following TBI (Dent et al., 2015; Shi et al., 2015). Several factors contribute to oligodendrocyte death and axonal demyelination, including glutamate and calcium cytotoxicity, oxidative stress, pro-inflammatory cytokine release by microglia, and the loss of crosstalk with astrocytes and neurons (Matute, 2011; Shi et al., 2015). Hence, finding approaches to prevent oligodendrocyte death or to enhance oligodendrocyte progenitor cell (OPC) maturation and remyelination will provide novel means to restore WM integrity and improve neurological recovery in TBI patients. Some of the key molecules under investigation to enhance oligodendrocyte survival and diminish axonal demyelination are the ionotropic (P2X) and metabotropic (P2Y) purinergic receptor families (Welsh and Kucenas, 2018). Extracellular nucleotides such as ATP participate in several physiological processes *via* purinergic receptors: neurotransmission and neuromodulation, regulation of the activity for glial cells (microglia and astrocytes), and axonal myelination by oligodendrocytes. However, the same receptors mediate neurodegeneration and demyelination when, under stressful conditions, damaged cells release increased amounts of nucleotides into the extracellular space, evoking excitotoxic degeneration (Puchałowicz et al., 2014). Namely, ATP released in significant amounts chronically activates calcium-permeable P2X7 purinergic receptors, which are highly expressed on the surface of differentiated and mature oligodendrocytes, leading to oligodendrocyte death, demyelination, and axonal injury. Notably, P2X7 receptor antagonists have been shown to prevent ATP-mediated excitotoxicity in oligodendrocytes and to inhibit demyelination by countering the P2X7-facilitated intracellular calcium elevation triggered by ATP (Matute et al., 2007). Moreover, P2Y receptor activation was shown to be involved in the control of migration and maturation of OPCs (Agresti et al., 2005). As shown by Agresti and collaborators, ATP and ADP inhibit the proliferation of OPCs induced by platelet-derived growth factor while inducing OPC migration *via* the activation of the P2Y_1_ receptor, the main metabotropic receptor expressed in OPCs, whose effects can be dampened by the presence of the P2Y_1_ antagonist MRS2179. Given these findings, expanding our understanding of the roles of these receptors will increase the likelihood of successes for pharmacological therapies focused on OPCs migration and maturation, and myelin regeneration. Studies with hESC-derived OPCs have demonstrated differential expression patterns and effects from the modulation of P2X and P2Y receptors during OPC maturation (Kashfi et al., 2017). In this regard, *in vitro* cultures of oligodendrocytes differentiated from hESCs or iPSCs (Wang et al., 2013a; Douvaras et al., 2014; Douvaras and Fossati, 2015; Ehrlich et al., 2017) represent a valuable model for identifying optimal pharmacological targets for the prevention of oligodendrocyte degeneration.

WM is highly susceptible to TBI-related ischemia. Lowering of the blood supply initiates small vessel remodeling within WM fibers during which endothelial tight junctions degenerate and allow serum molecules to penetrate the brain (Rosenberg, 2009). Specifically, fibrinogen extravasation initiates a cascade of chronic inflammation by activating microglia (Ryu and McLarnon, 2009; Davalos et al., 2012), which has detrimental effects on OPCs and oligodendrocyte survival (Yune et al., 2007; Pang et al., 2010; Li et al., 2017). The tight dependence of WM on the vascular system requires that reparative therapies integrate a multifactorial approach that counters endothelial dysfunction and inflammation, since oligodendrogenesis may not be sufficient to fully restore WM fibers (Hamanaka et al., 2018). Indeed, transplantation of hiPSC-derived endothelial cells (ECs) within demyelinated areas has been shown to form functional vessels in a mouse model of WM ischemic infarct (Xu et al., 2019). Transplantation of ECs enhanced cell survival, increased the number of OPCs, suppressed inflammatory responses and astrocytosis, decreased the ischemic area, promoted remyelination, and recovered limb coordination. Taken together, these results suggest that EC transplantation accelerates WM recovery after TBI.

### Cerebrovascular Dysfunction and Repair

In parallel with axonal damage, in the acute phase, shear stress causes brain vessel disruption and vascular dysfunction that encompasses changes in the blood-brain barrier (BBB), microhemorrhages, focal ischemia, and edema (Logsdon et al., 2015). Namely, BBB disruption in this phase induces calcium perturbations within cells that trigger cellular stress, inflammation, and apoptosis. However, it is the delayed microvascular pathology that is associated with prolonged inflammation, WM degeneration, long-term neurodegeneration, and disability (Glushakova et al., 2014; Sandsmark et al., 2019). Microvascular pathology has been proposed as a link between TBI and the greater prevalence of Alzheimer’s disease-like pathology and dementia in these patients (reviewed in Ramos-Cejudo et al., 2018). Vascular dysfunction appears to be related to the appearance of several major histological hallmarks of AD in TBI patients. On one hand, it impedes amyloid β (Aβ) clearance, thus favoring perivascular aggregation and Aβ-mediated oxidative stress, endothelial dysfunction, and death worsening the chronic TBI-driven encephalopathy. On the other hand, the suppression of nitric oxide production by impaired ECs is associated with increased ratios of tau phosphorylation. Hence, the full recovery of the cerebrovascular function is essential to maintain brain homeostasis and to palliate TBI-derived pathology long term.

The receptor for advanced glycation end-products (RAGE) is involved in BBB and WM fiber degeneration following intracerebral hemorrhages. Consequently, RAGE antagonists have been proposed for therapeutic intervention to prevent hemorrhage-related injuries and microgliosis (Yang et al., 2015). According to the study by Yang et al. (2015), in which they injected autologous arterial blood into the basal ganglia to recreate intracerebral hemorrhages, the iron released by the degenerating hemoglobin exacerbates RAGE expression, mainly in microglia, and initiates a RAGE-dependent signaling cascade leading to increased BBB permeability and WM fiber degeneration. The blockage of this response with a RAGE antagonist (FPS-ZM1) exhibited considerable benefits in terms of reduced BBB permeability, brain edema, motor dysfunction, and nerve fiber injury, as well as reduced expression of proinflammatory mediators.

Due to species differences in BBB receptor expression (Warren et al., 2009; Song et al., 2020) and the limitations in acquiring fresh vascular tissue from human biopsies; vascular research can benefit from hiPSC-derived BBB *in vitro* models. hPSC-derived ECs co-cultured with astrocytes respond to astrocytic cues, express a variety of endothelial transporters and receptors, and recapitulate several relevant BBB attributes including well-organized tight junctions and polarized efflux transporter activity (Lippmann et al., 2012). Thus, *in vitro* BBB-like cultures are important tools for understanding BBB pathology. However, these models remain challenging since EC function is highly dependent on blood pressure-induced shear stress (Thosar et al., 2012), and EC cultures in a dish do not mimic the complex vascular physiology of the BBB. In particular, they cannot self-assemble into vascular networks and fail to form a functional vasculature. Fabricating microfluidic channels based on biomaterials that can be further endothelialized with human umbilical vein ECs or iPSC-ECs is a promising approach (Williams and Wu, 2019). In this regard, hiPSC-derived BBB chips enable more reliable disease modeling since they incorporate flow dynamics and facilitate BBB-brain tissue interactions by combining two or more iPSC-derived cell types in the same chip (Vatine et al., 2019). Nevertheless, one of the major current challenges is to reduce the microchannel diameter to better mimic physiological vessel diameters and improve tissue-engineered microvascular networks (Williams and Wu, 2019). Regarding vascular function recovery *in vivo*, a potential strategy for restoring perfusion in ischemic tissue is to apply autologous hiPSC-derived ECs, alone or in combination with a printed scaffold, directly into the affected area to replace the dysfunctional vasculature and promote the growth of new blood vessels (Rosa et al., 2019). However, vascular function is not limited to ECs but requires the proper functioning of several cells that comprise the expanded neurovascular coupling (eNVC): neurons, astrocytes, endothelial cells, pericytes, and smooth muscle cells (reviewed in Salehi et al., 2017). Often, after the structural recovery of the brain vessel following a traumatic event, the eNVC function is not fully restored. Thus, another challenge for *in vitro* models of the vascular system is to recapitulate the complexity of the eNVC and permit the study of more complex cell-to-cell interactions beyond the structural recovery.

## Inflammatory Response in TBI

The axonal shearing, vascular disruption, and ischemia associated with TBI pathophysiology elicits a complex multi-stage immune response, which can be neuroprotective in some instances and neurotoxic in others (Loane and Kumar, 2016). Mechanical injury leads to the release of damage-associated molecular patterns (DAMPs), including alarmins and pathogen-associated molecular patterns (PAMPs). Among these, Galectin-3 (Gal-3) functions as a crucial regulator of the inflammatory response (Simon et al., 2017; Yip et al., 2017). These signals lead to microglial cell recruitment and activation at the site of damage. Microglia are among the earliest immune effectors and undergo polarization along a spectrum ranging from M1-like to M2-like phenotypes (Simon et al., 2017). The function of microglia includes cytokine, chemokine, and neurotrophin release, as well as debris phagocytosis (Jassam et al., 2017). The involvement of microglia-derived cytokines such as tumor necrosis factor-alpha (TNFα), IL-6, and IL-1β as signal-transducers that amplify inflammatory immune responses after trauma is well established (Simon et al., 2017). Notably, treatment with antibodies against Gal-3 has been shown to reduce cytokine release and neurodegeneration (Yip et al., 2017). Also, microglial depletion with the colony-stimulating factor 1 receptor (CSF1R) inhibitor Plexxikon 5622 was demonstrated to reduce TBI-induced neuroinflammation and neuronal cell death, leading to improved motor and cognitive function (Henry et al., 2020). Moreover, microglia-released pro-nerve growth factor (proNGF) was shown to promote oligodendrocyte death by binding the p75NTR receptor, thereby exacerbating neurodegeneration. This process can be countered by inhibiting proNGF release with minocycline (Yune et al., 2007). The modulation of microglial activation and function has been an extensively studied target for therapeutic intervention in TBI (Chio et al., 2015).

Importantly, crosstalk between activated microglia and other central nervous system-resident cells, such as astrocytes and peripheral immune cells, plays a crucial role in TBI-induced neuroinflammation. Astrocytes undergo reactive astrogliosis, upregulate the marker GFAP, and produce cytokines and chemokines (Simon et al., 2017). A recent report demonstrated that a member of the purinergic receptor family P2Y_1_ mediates the intercommunication between microglia and astrocytes in a mouse model of TBI (Shinozaki et al., 2017). Either ablation of microglial function or pharmacological inhibition of the P2Y_1_ receptor led to an altered reactive astrocyte response. Interestingly, knockout of the P2Y_1_ receptor enhanced the protective facet of astrogliosis and decreased neuronal damage in a mouse model of TBI. Furthermore, an elegant *in vivo* study of apoptotic neurons demonstrated that microglia and astrocytes act in a specialized but coordinated manner to effect clearance (Damisah et al., 2020). In particular, microglia were shown to encapsulate the soma of apoptotic neurons, while astrocytes polarized towards neurites. Robust activation of microglia and astrocytes is beneficial in targeting damaged neurons, but can also have toxic effects (Liddelow and Barres, 2017). Thus, understanding the regulation of the delicate balance between the neuroprotective and the neurotoxic potential of immune responses in TBI is important for the identification of new biomarkers and targets for promoting repair. Although rodents, especially mice, have been widely used to model neuroinflammation in developing pharmacological therapies, significant discrepancies in immune receptors, cell types, and signaling pathways between humans and mice may be responsible for the limitations of these models for drug discovery (Kodamullil et al., 2017). For this reason, *in vitro* models in human genetic backgrounds will help to complement drug screening studies towards validating the benefits and efficacy of potential new pharmacological therapies.

Recent protocols have shown efficient derivation of microglia-like cells from hiPSCs (Muffat et al., 2016; McQuade et al., 2018), as confirmed by expression of the microglial markers IBA1, CD45, and CD11B (reviewed in Hasselmann and Blurton-Jones, 2020). A study by Ormel et al. (2018) demonstrated that microglia can be grown within cerebral organoids (CO), representing the development of a valuable tool for studying the interactions of microglia with astrocytes, oligodendrocytes and neurons *in vitro*. COs are iPSC-derived three-dimensional (3D) cell cultures that recapitulate many of the features observed in a developing human brain in terms of cell types and cytoarchitectures and offer promise as an *in vitro* model of human brain diseases due to their ability to mimic complex interactions among the multiple cell types of the brain tissue (Lancaster et al., 2013; Lancaster and Knoblich, 2014). Human brain organoids enriched with microglia can serve as a window to study the multicellular implications of the neuroinflammatory response that follows traumatic axonal injury with a higher level of complexity than reported 2D primary tri-cultures of neurons, microglia, and astrocytes (Goshi et al., 2020). However, to date, *in vitro* models of inflammation do not fully capture the complex responses that follow TBI, as some major inflammatory process are driven by the infiltration of peripheral immune cells (reviewed in Reis et al., 2017). For instance, the infiltration of neutrophils alters the vascular permeability and contributes to oxidative stress and changes in cerebral blood flow that accentuates brain damage. On the other hand, infiltrating macrophages alternate between phagocytic, proteolytic, and proinflammatory states in the very early stages of anti-inflammatory and regenerative functions, including growth, angiogenesis, and matrix deposition at later stages.

## Neurogenesis and Stem Cell Therapies

There is currently no existing treatment capable of fully repairing the damage resulting from TBI and its sequelae (Zibara et al., 2019; Schepici et al., 2020). As outlined in previous sections, effective approaches to repair TBI-induced damage would promote not only neurogenesis, but also repair lost circuitry, integrate glial support cells, and form functional vasculature (Rolfe and Sun, 2015; Schepici et al., 2020), an extremely complex task that requires highly sophisticated strategies. Human SCs can proliferate and differentiate into all the cell types needed to regenerate injured brain tissue and have the potential to heal TBI-induced damage in ways that have not been possible with any other treatment to date (Skardelly et al., 2011; Zibara et al., 2019; Schepici et al., 2020). Numerous studies spanning more than two decades have tested the use of SCs for the treatment of TBI in animal models with varying degrees of success (Zibara et al., 2019). However, the translation of these approaches to the clinic is still at an early stage.

Regenerative therapies being currently explored focus on promoting repair either by stimulating endogenous SCs or by employing exogenous SCs (Kochanek et al., 2015; Rolfe and Sun, 2015; Zibara et al., 2019). The regenerative potential of SCs in the field of TBI has been widely reviewed before (Harting et al., 2008; Gennai et al., 2015; Reis et al., 2017; Zibara et al., 2019; Schepici et al., 2020). Here, we provide a summary of the main achievements and limitations of these therapies, to frame the current situation, before arguing the benefits of incorporating new biotechnological tools including hiPSC-derived three-dimensional (3D) cell cultures and biomaterials to enhance the success of reparative strategies for TBI patients.

### Stimulating Endogenous Stem Cells

Endogenous-targeted regenerative therapies aim to accomplish repair by stimulating local neurogenesis and other endogenous restorative processes. Given that regeneration and functional recovery remain major challenges in TBI, relying on endogenous neurogenesis alone is not sufficient, and therapeutic approaches are necessary to augment the body’s response. These therapeutic strategies are designed to capitalize on resident neural SCs and rely on the presence of these cells to be effective. It is worth noting that although there are cells with neurogenic and gliogenic potential in the adult human brain, the extent of neurogenesis in adulthood is controversial (Eriksson et al., 1998; Rakic, 2004; Sanai et al., 2004; Spalding et al., 2013; Sun, 2016; Boldrini et al., 2018; Lee and Thuret, 2018; Paredes et al., 2018; Sorrells et al., 2018). However, in the context of ischemia or TBI, research points to the activation of neurogenesis and gliogenesis following injury (Sun, 2016; Zibara et al., 2019). As reported in the study by Zheng et al. (2013), in TBI patients, the expression of neural stem/progenitor cell markers including DCX, TUC4, PSA-NCAM, SOX2, and NeuroD was increased in the perilesional cortex compared to a normal brain. Also, the cell proliferation marker, Ki67, was significantly increased within the affected area and colocalized with neural progenitor markers suggesting trauma-driven neurogenesis (Zheng et al., 2013). These types of observations have promoted further research into potentiating endogenous neurogenesis after TBI events.

Endogenous-targeted therapeutic approaches aim to promote endogenous stem cell migration, proliferation, survival, differentiation, integration, or maturation. Potential molecular effectors that act on SCs include morphogens, hormones, growth, and neurotrophic factors, and these have garnered interest as potential therapeutics (Lledo et al., 2006; Faigle and Song, 2013; Mouhieddine et al., 2014; Berninger and Jessberger, 2016; Zibara et al., 2019). Numerous small molecules have already been tested for their capacity to make the environment more conducive to regeneration. Among these are compounds that target oxidative stress, the inflammatory response, neurodegeneration, and apoptosis, including nimodipine, selfotel, and cyclosporine. Other small molecules, such as progesterone and erythropoietin, are purported to ameliorate the damaged environment through neuroprotective, neurotrophic, or angiogenic properties (reviewed in Zibara et al., 2019). Although many drugs showed considerable promise in animal models, most failed to produce functional improvement in clinical trials (Kochanek et al., 2015; Zibara et al., 2019). Despite over 400 clinical trials, there are currently no approved drugs that can modulate endogenous SCs for the treatment of TBI, and major challenges remain to be addressed if endogenous SCs are to enable effective recovery; these include insufficient neurogenesis, inadequate neuronal differentiation, and maturation, and low survival rates (Zibara et al., 2019). However, some promising results have been obtained with exogenous stem cell transplantation.

### Transplanting Exogenous Stem Cells

Transplanted exogenous SCs can promote repair in two ways. They can directly give rise to new neurons and glia to regenerate the damaged tissue, or they can promote repair *via* a bystander effect (Rolfe and Sun, 2015; Napoli et al., 2018). The latter stimulates the endogenous neurogenic niche and innate repair mechanisms through the secretion of various molecules. For example, exogenous SCs can secrete growth factors for trophic support, cytokines, extracellular matrix (ECM) molecules, and exosomes, which have been shown to reduce the inflammatory response, mediate neuroprotection, and stimulate endogenous stem cell activation and neurogenesis (Tajiri et al., 2014; Kochanek et al., 2015; Rolfe and Sun, 2015; Napoli and Borlongan, 2017; Zuo et al., 2017; Schepici et al., 2020). In this regard, the bystander mechanism of repair shares similarities with the endogenous stem cell-based approach for TBI treatment but depends on cell transplantation to stimulate neurogenesis. Of note, transplanted SCs engineered to express neurotrophic factors have been demonstrated to further enhance this potential (Bakshi et al., 2006; Blaya et al., 2015; Chen et al., 2017). Neurotrophic factors regulate neuronal survival by promoting differentiation, neurite outgrowth, synaptic plasticity, cell repair, or apoptosis, however, the efficiency of neurotrophin-based treatments is compromised by their short half-lives, reduced BBB permeability, and the limited diffusion within the neural parenchyma. Also, the fact that cells express neurotrophin Trk (tropomyosin-related kinase) receptors differentially limits the broad therapeutic effect of these molecules. In this sense, providing multifunctional neurotrophins could enhance its benefits. For example, in the study by Blaya et al. (2015) in a rat model of TBI, the pericontusional transplantation of neural progenitors, collected from rat fetuses and genetically modified to secrete a synthetic human multifunctional neurotrophin (MNTS1), reported long term benefits regarding transplant survival and neuronal differentiation. However, the secretion of MNTS1 did not have an impact on the cytoarchitecture preservation compared to animals transplanted with non-genetically modified neuronal progenitor and did not significantly improve the hippocampal-dependent learning and memory performance in these transplanted animals. Moreover, SCs transplanted into the brain have also been found to create a “bio bridge” between the injury site and the neurogenic niche for migration of neurogenic cells to the lesion (Tajiri et al., 2013, 2014). Taken together, studies using exogenous SCs for TBI treatment have shown encouraging results, and point to a prominent bystander role, although further study is required (Napoli and Borlongan, 2017; Zuo et al., 2017; Napoli et al., 2018).

A variety of different types of SCs have been explored for TBI treatment in animal models, including embryonic SCs, adult neural SCs, and different types of mesenchymal SCs, such as bone marrow SCs, amnion-derived multipotent progenitor cells, adipose-derived SCs and umbilical cord-derived SCs (Rolfe and Sun, 2015; Sun, 2016; Zibara et al., 2019; Schepici et al., 2020). Even hiPSCs directly derived from the fibroblasts of the dura mater of TBI patients that undergo surgery have been proposed as a new source of SCs to generate neuronal progenitor cells (Cary et al., 2015). The specific mechanisms of brain tissue repair facilitated by different stem cell therapies have been recently reviewed (Zhou et al., 2019). The most common methods of administration are intravenous or stereotactic injection into the brain (Schepici et al., 2020). Briefly, the results of animal studies have collectively demonstrated survival and migration of the transplanted cells, and differentiation of SCs into neurons, astrocytes, and oligodendrocytes; they have shown increased angiogenesis, reduced astrogliosis, and lesion volume, and attenuated axonal degeneration; and, also, many have reported accompanying improvements in cognitive and motor functions (reviewed in Schepici et al., 2020). Nonetheless, other studies have failed to rescue cognitive function despite the reduced lesion size observed in injured brains that received stem cell treatment and the successful recovery of the motor function in these individuals, suggesting that a complex function like memory may be more challenging to recover (Hoane et al., 2004). Animal models have also demonstrated that intravenous or intra-arterial injected SCs preferably migrate to the damaged region and that the microenvironment of the trauma drives the transplanted SCs to a neural phenotype, but the fate of those SCs that migrate to non-target organs (e.g., lung and liver) is unclear (Lu et al., 2001, 2002) and this issue is still a big concern in the field. More recently, intravascular injection of stem cell-derived exosomes alone have proven beneficial in promoting angiogenesis and neurogenesis and in reducing hippocampal neuronal cell loss by drastically reducing neuroinflammation, leading to the notion of exosomes as a novel cell-free therapy for TBI (Zhang et al., 2015, 2020). Exosomes are small extracellular vesicles, 30–100 nm in diameter, involved in the cell-to-cell communication through the transport of various RNAs and proteins such as immunoinhibitory proteins with therapeutic benefits (Zhou et al., 2019). These cellular components, derived from multiple cell types, have been used in the diagnosis of some pathologies associated with cancer, genetics, or pathogenic infections, and are now under investigation for their therapeutical potential due to their low immunity, long half-life in the peripheral circulation, and their ability to cross the BBB.

Beyond animal work, six clinical trials have been completed to date, all using bone marrow-derived SCs except one, which used umbilical cord-derived SCs (Cox et al., 2011, 2017; Tian et al., 2013; Wang et al., 2013b; Liao et al., 2015; Cramer et al., 2019). In the most recent trial, lead by Cramer et al. (2019), 61 chronic TBI patients were randomized into stem cell treated groups [receiving three different doses of allogeneic modified bone marrow-derived mesenchymal SCs (SB623)] by intracranial injection or surgical sham. The study showed no dose-limiting toxicities or deaths in treated groups and a statistically significant improvement in the Fugl–Meyer Motor Scale score at 24 weeks post-treatment, and the group treated with the highest dose (5.0 × 106 SB623) was the one showing the greatest benefits in motor status. In general, all six trials demonstrated safe use and some demonstrated improvements in motor and cognitive functions, as well as reduced inflammation. Although the completed trials presented encouraging results, the size of patient cohorts in the studies was small, making it difficult to draw conclusions in the absence of larger patient numbers from multicentric collaborations, to confirm the benefits of these stem cell transplantation approaches. Six ongoing trials (listed on clinicaltrials.gov) will use bone marrow or adipose-derived SCs to treat larger numbers of patients, and will provide further evidence as to the efficacy of exogenous stem cell therapy in TBI (Schepici et al., 2020).

Despite the encouraging results in preclinical and clinical studies, stem cell therapies are certainly not considered miracle treatments as the field stands currently, and there are several issues to be addressed going forwards. The success of stem cell therapies for TBI treatment in the clinic will depend on improving multiple parameters. In the case of endogenous stem cell therapy, the focus is on developing better strategies to guide the migration of endogenous stem cell-derived new neurons to the injured site and on promoting vascularization and cell survival. In the case of exogenous stem cell transplants, reducing the risk of undesired side effects such as seizures or tumor formation (Dekmak et al., 2018) and enhancing transplant viability by countering the hostility of the host environment are current priorities (Weston and Sun, 2018). One aspect of the latter is the need to overcome the immunogenic and rejection-prone nature of autologous stem cell grafts. The identification of mitochondrial DNA (mtDNA)-derived neoantigens and characterization of the immune responses they elicit following autologous iPSC grafts represents a major step forward in this direction (Deuse et al., 2019). Specifically, Deuse et al. showed that subcutaneous transplantation of high passage iPSCs with single nucleotide polymorphisms (SNPs) in mtDNA that give rise to neoantigens, led to decreased survival of grafted cells and elicited a strong T cell response associated with IFN**γ** and IL-4 production in mice. Of note, these features were not unique to iPSC grafts but were also mirrored by differentiated endothelial cell grafts, which implies persistent immunogenicity across cell differentiation. These findings demonstrate that autologous iPSCs should be screened for neoantigenic SNPs before transplantation to avoid immune rejection. Other facets of improving transplant viability can be addressed through the use of scaffolding support constructs, which enhance stem cell grafting and survival, or by directly transplanting 3D cell structures from patient-derived iPSCs, such as brain organoids or bio-printed neural tissue on biocompatible scaffolds.

### Tissue Engineering Strategies

The 3D engineered tissue transplant can contain a complete microenvironment, including neuronal progenitors, mature neurons, astrocytes, and oligodendrocytes (Lancaster and Knoblich, 2014; Kim et al., 2019). These structures are thought to enhance cell adaptation and survival in the injured area as they facilitate vascularization in the transplanted area (Mansour et al., 2018). Potentiating vascularization ensures that the nutrient and oxygen supply reaches the area to be repaired and promotes cell survival, maturation, and functional recovery (Ong et al., 2018; Wang et al., 2020). As reported by Wang et al. (2020) in a model of motor cortex injury in rats, transplanted COs enhanced brain repair and were successfully vascularized. Interestingly, improved results were obtained when they used less mature COs (50 days post differentiation) in comparison with more mature ones (85 days post differentiation). This observation would imply that a greater number of neuronal progenitors in the COs could be more beneficial for tissue restoration. Similarly, pre-vascularizing bio-printed tissues with hiPSC-derived ECs or incorporating angiogenic molecules into bio-printed tissues enhanced vascularization from the host (Moon and West, 2008). Moreover, engineered tissues are of particular interest since they offer the possibility of being generated in a way that fulfills injury-specific requirements regarding shape, size, and cell type content for each individual transplant. In preparation for customizing engineered tissues, the damaged area can be scanned with magnetic resonance imaging and a 3D reconstruction of the affected volume can be used to optimize the modeling of the support scaffold (Fu et al., 2017). On the undesirable side, hiPSC-derived tissues are believed to present a higher risk of tumorigenicity due to the reprogramming process by viral infection and so these stem cell sources has not been yet considered for clinical applications (Zhou et al., 2019).

**Figure 1:**
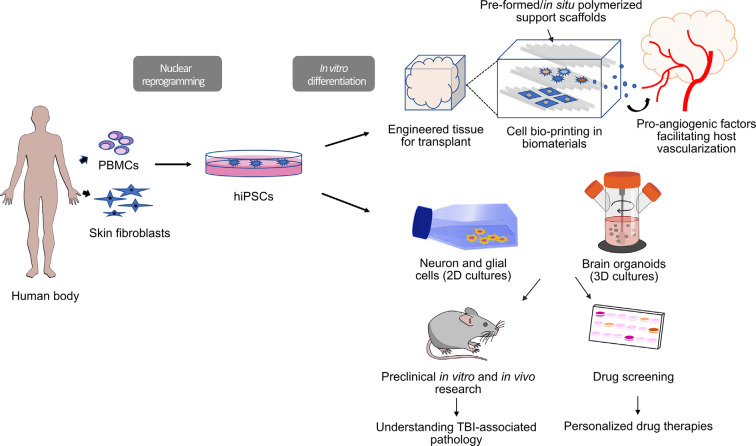
Workflow for human induced pluripotent stem cell (hiPSC)-derived tools for clinical and preclinical research in traumatic brain injury (TBI). HiPSCs are induced from fibroblasts (skin biopsy) or peripheral blood mononuclear cells (PBMC; blood collection). Three-dimensional (3D) constructs are engineered *via* bio-printing in biocompatible materials using hiPSC-derived cultured cells (bioinks). Bio-printed tissues can be enriched with pro-angiogenic molecules to enhance host vascularization after transplant. These 3D constructs could potentially be used for transplantation as part of regenerative therapies. Alternatively, hiPSC-derived cells can be grown as 2D and 3D cultures (brain organoids) that can be used for drug screening or *in vitro* disease modeling.

In addition to the potential benefits that engineered tissues could have for tissue regeneration, these 3D cultures also represent a useful tool for drug screening and for the study of the very first physiological alterations that occur in traumatic events (Figure 1). Mimicking the cerebral cortex in a dish makes it possible to perform functional assessments in real-time of physiopathological events that take place in the acute phases of the traumatic event (e.g., injury-induced glutamate release and transient hyperactivity) that would not be possible to monitor in humans or animal models due to obvious time constraints (Shuler and Hickman, 2014; Tang-Schomer et al., 2014; Chwalek et al., 2015). Namely, these types of tissues could be of great interest for understanding glutamate release-mediated excitotoxicity in TBI patients and the screening of anti-excitotoxicity molecules. Acute excitotoxicity is mainly caused by impact-induced presynaptic glutamate release acting on AMPA and NMDA receptors but, to date, treatments available for countering its effects hinder neuronal survival and worsen TBI outcomes (Kochanek et al., 2015). In comparison with 2D cultures, brain organoids and bio-printed tissues also have the advantage of more accurately recapitulating the cell-to-ECM interactions, which has been proven a key modulator of drug efficiency (reviewed in Langhans, 2018).

While biomaterial scaffolds can be used as components of pre-formed engineered tissues, they can also be introduced into the injured area in the brain to help create a pro-regenerative environment and support tissue regeneration *in situ* (Khaing et al., 2014; George and Steinberg, 2015; Boisserand et al., 2016; Nih et al., 2016; González-Nieto et al., 2018; Gopalakrishnan et al., 2019). This is a strategy that has been largely unexplored in the context of TBI treatment but that could have extensive benefits. Certain biomaterials like collagen and hyaluronic acid can be polymerized *in situ* to form hydrogel matrices and are compatible with stereotactic injection into the brain. These materials are injected in liquid form, thus they can fill and conform to the site of injury, and subsequently polymerize into a gel with mechanical properties similar to brain tissue (Mekhail and Tabrizian, 2014; Boisserand et al., 2016; Nih et al., 2016). The incorporation of biomaterials is a strategy that can complement both the endogenous and exogenous stem cell transplantation approaches, as a structural support scaffold for cells at the injury site, and as a delivery vehicle for small molecule therapeutics (Lu et al., 2007; Walker et al., 2009; Khaing et al., 2014; Boisserand et al., 2016).

Using biomaterial scaffolds in stem cell-based TBI treatments could provide many benefits by facilitating stem cell migration and survival, and by reducing the unfavorable host conditions. Hydrogel matrices can be prepared from many different molecules, including numerous proteins and polysaccharides; when the material polymerizes from a liquid to a gel, long molecules link together into a mesh structure, forming an ECM-like scaffold (Tibbitt and Anseth, 2009; Somaa et al., 2017). This structure provides 3D support for cell infiltration and attachment, enabling cells to migrate into the area, providing them with a physical structure to adhere to so they can remain there. In the context of ischemic brain damage, biomaterial scaffolds have indeed been found to promote migration and survival of both endogenous and transplanted neural cells, and to promote their proliferation and differentiation (Lam et al., 2014; Moshayedi et al., 2016; Nih et al., 2017; Somaa et al., 2017). In addition to neural cells, these benefits apply to ECs, as biomaterials have been found to promote angiogenesis and revascularization of brain lesions (Ju et al., 2014; Nih et al., 2018). Functional vasculature is critical for nutrient and oxygen supply to the damaged area, and as has been highlighted, restoring this function is essential to support regeneration following TBI. Moreover, biomaterials can be modified to contain specific cell adhesion motifs to further encourage cell infiltration and attachment (Cui et al., 2006; Lampe et al., 2013; Moshayedi et al., 2016). The RGD motif from fibronectin is often used, and the concentration of motifs in the material can be tailored. Optimized motif concentrations have also been shown to promote NPC survival, neuronal differentiation (Moshayedi et al., 2016), and neurite extension (Cui et al., 2006), so there are numerous potential benefits to these engineered materials.

Furthermore, certain biomaterials can interact with immune cells, reducing inflammation (Austin et al., 2012; Nih et al., 2018) and glial scarring at the injury site, and are considered immunomodulatory (Hou et al., 2005; Khaing et al., 2011; Nih et al., 2017). For instance, high molecular weight hyaluronic acid has been shown to decrease microglial inflammatory signaling (Austin et al., 2012). In addition to physical supports, biomaterials can provide trophic support by acting as a depot for soluble factors either delivered with the biomaterial (Wang et al., 2012; Cook et al., 2017; Nih et al., 2018) or secreted by infiltrating cells, thereby maintaining a high concentration of those factors where they are most needed (Pakulska et al., 2012; Khaing et al., 2014; George and Steinberg, 2015; Boisserand et al., 2016; Nih et al., 2016, 2018; González-Nieto et al., 2018; Gopalakrishnan et al., 2019). In general, biomaterials have garnered significant interest as drug delivery vehicles, for their abilities to provide local, controlled release of a therapeutic over time. This is of particular relevance to the brain, as the BBB poses a major challenge to intravenous drug delivery, and the prolonged release of drugs by a biomaterial platform provides sustained treatment in a single application/injection. This ability could be employed in TBI treatment to deliver anti-inflammatory or neurotrophic factors and help create a pro-regenerative environment. Also, these materials can be biodegradable or bioresorbable to allow for their gradual replacement with new tissue (Pakulska et al., 2012; Nih et al., 2016; González-Nieto et al., 2018). These properties have proven to be important for successful regeneration and ultimately result in the elimination of any foreign material, leaving only the tissue behind.

As a relevant example, biomaterials have been employed in numerous studies to promote regeneration in the stroke-damaged brain, where the injury microenvironment is as hostile as it is in TBI. Other issues faced by stroke therapies that are pertinent to TBI include the dispersion away from the transplantation site and low survival rates of exogenous SCs, the low numbers of migrating endogenous SCs, and the low levels of differentiation and integration with existing tissue. These issues are shared by TBI stem cell therapies, and attempts are being made in the context of stroke to address all of them *via* the incorporation of biomaterials (Nih et al., 2016; González-Nieto et al., 2018; Gopalakrishnan et al., 2019; Ho et al., 2019; Obermeyer et al., 2019). Biomaterials have been used in stroke treatments as scaffolds to support endogenous cell infiltration (Ghuman et al., 2016, 2017; Nih et al., 2017), and as delivery vehicles for small molecules (Wang et al., 2012; Cook et al., 2017; Nih et al., 2018) and SCs with encouraging results (Moshayedi and Carmichael, 2013; Lam et al., 2014; Moshayedi et al., 2016; Nih et al., 2018; Payne et al., 2019).

The importance of physical factors like support scaffolds for cells has thus far been largely underexplored in the TBI field. Several animal studies that used biomaterial scaffolds in combination with SCs for TBI treatment demonstrated beneficial effects. They have tested chitosan microspheres, collagen, collagen-fibronectin, and collagen-hyaluronate scaffolds, some functionalized with various moieties, to deliver neural progenitor or other SCs into the brain. Notable effects included improved survival and migration of cells injected with the biomaterial (Tate et al., 2002; Skop et al., 2016), and differentiation into glia, vascular endothelial cells (Elias and Spector, 2012), and neurons (Shi et al., 2016). Intriguingly, several studies found improvements in neuropathological parameters, such as reduced lesion volume and axonal degeneration, increased angiogenesis, infiltration of endogenous neural progenitor cells, and differentiation into neurons with the formation of functional synapses, as well as improvements in motor and cognitive functions, such as spatial learning and sensorimotor function (Lu et al., 2007; Xiong et al., 2009; Chen et al., 2011; Duan et al., 2016). Although the majority of these studies used collagen-based scaffolds, there are many biomaterials to choose from, and hyaluronic acid is of particular interest for stroke stem cell therapies (Moshayedi and Carmichael, 2013; Lam et al., 2014; Moshayedi et al., 2016; Nih et al., 2017; Ho et al., 2019). The promising results of this small number of studies using scaffolds together with stem cell treatment are encouraging, but biomaterials are missing from the majority of animal studies and all clinical trials for TBI treatment. We believe that further attention to biomaterials and their incorporation into future experiments and clinical trials will be crucial for the improvement of outcomes of stem cell-based therapies for TBI and the development of the field.

## Conclusion and Perspectives

SCs are valuable tools for modeling and understanding the cellular and molecular mechanisms of neurodegeneration and repair in TBI. Namely, hiPSC-derived 2D or 3D cell cultures provide researchers with appropriate models for drug screening in a human genetic background, thereby representing a significant step forward towards personalized medicine. However, these *in vitro* models have their limitations, that include not incorporating some of the relevant cell types from the peripheral immune system, a lack of a complex ECM that can modulate drug efficiency, and the lack of a functional BBB that could limit, *in vivo*, the success of the drugs that have been validated *in vitro* with these models.

Regarding regenerative therapies, while significant progress has been made in recent years towards the treatment of TBI using exogenous SCs, many challenges remain. Some issues include the low survival rates of exogenous SCs and their tendency to disperse away from the transplantation site, a fact that seems governed by the harsh environment within the injured area. Stem cell-derived 3D structures and biomaterials applied either jointly with exogenous SCs or alone, provide structural support and can help reproduce the necessary microenvironment for successful transplantation of cells or endogenous repair. For instance, optimizing scaffolds to better interact with transplanted SCs seems promising. Also, biomaterials have proven highly beneficial in restoring stroke-induced brain damage, and the results from the few preclinical studies that have applied this strategy to the TBI field are encouraging. In our view, the optimization of biomaterials in combination with a better understanding and modulation of the inflammatory response, and promotion of vascularization within the injured region represents the most important areas of focus for enhancing the success of regenerative therapies in TBI.

## Author Contributions

ML-A and CC conceived and wrote the manuscript. CZ participated in the writing of the manuscript. LB and TD edited and supervised the work.

## Conflict of Interest

The authors declare that the research was conducted in the absence of any commercial or financial relationships that could be construed as a potential conflict of interest.
